# AFB1 hepatocarcinogenesis is via lipid peroxidation that inhibits DNA repair, sensitizes mutation susceptibility and induces aldehyde-DNA adducts at p53 mutational hotspot codon 249

**DOI:** 10.18632/oncotarget.15313

**Published:** 2017-02-14

**Authors:** Mao-Wen Weng, Hyun-Wook Lee, Bongkun Choi, Hsiang-Tsui Wang, Yu Hu, Manju Mehta, Dhimant Desai, Shantu Amin, Yi Zheng, Moon-Shong Tang

**Affiliations:** ^1^ Departments of Environmental Medicine, Pathology and Medicine, New York University School of Medicine, Tuxedo, NY 10987, USA; ^2^ Department of Pharmacology, Penn State University College of Medicine, Hershey, PA 17033, USA

**Keywords:** aflatoxin B1, hepatocellular carcinoma, p53 codon 249 mutations, AFB1-8,9-epoxide-deoxyguanosine, cyclic α-methyl-γ-hydroxy-1, N^2^-propano-dG

## Abstract

Aflatoxin B1 (AFB1) contamination in the food chain is a major cause of hepatocellular carcinoma (HCC). More than 60% of AFB1 related HCC carry p53 codon 249 mutations but the causal mechanism remains unclear. We found that 1) AFB1 induces two types of DNA adducts in human hepatocytes, AFB1-8,9-epoxide-deoxyguanosine (AFB1-E-dG) induced by AFB1-E and cyclic α-methyl-γ-hydroxy-1,*N*^2^-propano-dG (meth-OH-PdG) induced by lipid peroxidation generated acetaldehyde (Acet) and crotonaldehyde (Cro); 2) the level of meth-OH-PdG is >30 fold higher than the level of AFB1-E-dG; 3) AFB1, Acet, and Cro, but not AFB1-E, preferentially induce DNA damage at codon 249; 4) methylation at –CpG- sites enhances meth-OH-PdG formation at codon 249; and 5) repair of meth-OH-PdG at codon 249 is poor. AFB1, Acet, and Cro can also inhibit DNA repair and enhance hepatocyte mutational sensitivity. We propose that AFB1-induced lipid peroxidation generated aldehydes contribute greatly to hepatocarcinogenesis and that sequence specificity of meth-OH-PdG formation and repair shape the codon 249 mutational hotspot.

## INTRODUCTION

Hepatocellular carcinoma (HCC) is the major cancer leading to death in developing countries [1]. The incidence of HCC in these countries correlates well with AFB1 contamination in the food chain [2]. AFB1 can cause HCC in rat models [3]; therefore, it is generally accepted that AFB1 is a major human HCC etiological agent [4]. The HCCs occurring in the AFB1 contaminated regions have a unique molecular feature, which is the high prevalence of p53 codon 249 -AGG- to -AGT- mutations (>60% of all p53 mutations) (Supplementary Figure 1) [5]. The prevalence of codon 249 mutations in HCC is linearly proportional to the levels of AFB1 food contamination [6].

p53 is a tumor suppressor gene frequently mutated in human cancers [7, 8]. p53 mutational patterns often bear the etiological fingerprints of carcinogens [9]: For example, p53 mutation hotspots in skin cancer are located at sequences containing contiguous pyrimidines that are the preferential sites for ultraviolet (UV) photodimer formation; p53 mutational hotspots in cigarette smoke (CS)-related lung cancer are located at cytosine methylated -CG- sites that are preferential binding locations for CS carcinogens such as the diol epoxide forms of polycyclic aromatic hydrocarbons (PAHs) and acrolein (Acr) [5, 10–13].

AFB1 is produced by *Aspergillus* *flavus* and related fungi. It is mainly metabolized in the liver and is the most hepatotoxic and carcinogenic of the aflatoxins [14]. In rat and human liver cells, AFB1 is biotransformed by cytochrome P450 (CYP) to a highly reactive intermediate, AFB1-8, 9-epoxide (AFB1-E) [15]. Although AFB1 itself is unable to react with DNA, AFB1-E can rapidly react with N7 of guanine residues to form the highly mutagenic bulky AFB1-E-deoxyguanosine (AFB1-E-dG) and AFB1-formamidopyrimidine (AFB1-FaPy)-dG adducts [15, 16]. AFB1 processing in liver cells also generates an extensive amount of reactive oxygen species (ROS) and initiates lipid peroxidation (LPO) through the extraction of a hydrogen atom from unsaturated fatty acids of membrane phospholipids [17, 18]. LPO byproducts, such as Acr, crotonaldehyde (Cro), acetaldehyde (Acet), and 4-hydroxy-2-nonenal (HNE), can also react with DNA to form highly mutagenic bulky cyclic propano-dG (PdG) adducts [19–22]. These findings raise two pertinent questions for AFB1 hepatocarcinogenesis: Is AFB1-E, the major AFB1 metabolite, or is it AFB1-induced secondary toxicants, aldehydes and ROS, that drive carcinogenesis? Which of these DNA damaging agents induce *p53* codon 249 mutations in hepatocytes? Is it AFB1-E, ROS, aldehydes, or a combination of a few or all the above? Understanding the molecular detail of these events will not only enhance our understanding of AFB1 hepatocarcinogenesis, but will also enable us to better assess the risks for liver cancer development and design effective prevention measures.

To address these questions, we measured DNA adducts induced by ROS, lipid peroxidation (LPO) aldehyde byproducts, and AFB1-E in AFB1 treated human hepatocytes. We found that >30 fold more cyclic α-methyl-γ-hydroxy-1,*N^2^*-propano-dG (meth-OH-PdG) adducts than AFB1-E-dG are formed in these cells. We also found that Acet and Cro can induce meth-OH-PdG in hepatocytes. We then mapped the DNA adduct distribution induced by AFB1, Cro, Acet, and AFB1-E in the p53 gene. We found that while AFB1-E-dG adducts are preferentially formed at codons containing –CpG- sequences and codon 249 is not included. In contrast, AFB1, as well as Acet and Cro, induce DNA adducts that are preferentially formed at codons containing –GG- including codon 249. AFB1, as well as Acet and Cro, treatment inhibits DNA repair and sensitizes mutational susceptibility of human hepatocytes. Thus, we proposed that AFB1 hepatocarcinogenesis occurs via the effects of aldehydes generated by the AFB1 metabolism induced LPO in hepatocytes including propagation of LPO cycle, induction of DNA damage and mutations at codon 249 of the p53 gene, inhibition of DNA repair, and, sensitization of hepatocytes’ susceptibility to DNA damage-induced mutagenesis.

## RESULTS

### AFB1-induced bulky DNA adducts in human HepG2 cells

**Figure 1:**
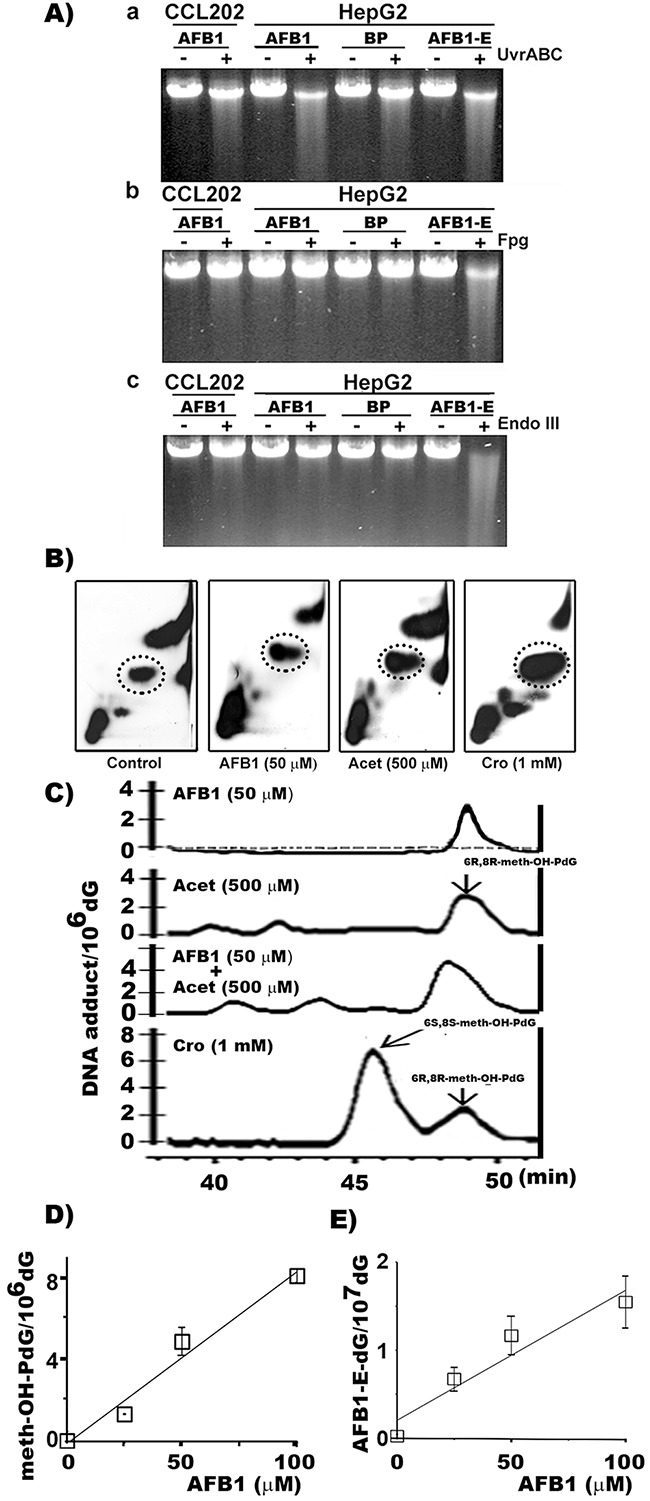
AFB1 induces >30 fold of meth-OH-PdG than AFB1-E-dG adducts in hepatocytes **A**. AFB1-induced DNA damage was first identified by DNA repair enzyme incision assay. Human hepatocytes (HepG2) and lung fibroblasts (CCL202 cells) were treated with AFB1 (100 μM, 6 h), benzo(a)pyrene (BP) (100 μM, 6 h), or AFB1-E (100 μM, 30 min). The bulky DNA adducts and oxidative DNA damage formed in the genomic DNA were detected by UvrABC, Fpg, and Endo III respectively, as described [26, 36, 59]. **B**. The cyclic propano-dG adducts induced by AFB1 in hepatocytes were then analyzed by the ^32^P post-labeling and the 2D-TLC/HPLC method [33, 34]. HepG2 cells were treated with: (1) AFB1 (50 μM for 6 h at 37 °C), (2) Acet (0.5 mM, 6 h at 37 °C), and (3) Cro (1 mM, 6 h at 37 °C). Shown are autoradiographs of typical ^32^P-post-labeling and 2D-TLC results. Materials in the circled spots were separated for HPLC analysis. **C**. Typical HPLC elution profiles resulted from Control (dotted line), AFB1, Acet, and Cro treated HepG2 cells. **D**. Levels of PdG and AFB1-E-dG formation in HePG2 cells treated with different concentrations of AFB1. PdG adducts were quantified by ^32^P post-labeling 2D-TLC/HPLC method and AFB1-E-dG adducts were quantified by competitive ELISA method [20, 57]. Note: (1) DNA adducts induced by AFB1 in HepG2 cells co-elute with Acet and Cro-induced 6R, 8R-meth-OH-PdG DNA adducts in HPLC analysis. (2) AFB1 induces PdG and AFB1-E-dG adducts at a 30-50 to 1 ratio. (3) AFB1 does not induce DNA damage in lung fibroblasts.

It is well established that ingested AFB1 is detoxified into AFB1-E by CYP1A2, 2D6, and CYP3A4 in liver in both human and rodent [23–25]. This AFB1 metabolic process also induces oxidative stress and LPO in hepatocytes [17, 18]. Therefore, it is possible that AFB1 can induce three types of DNA damage in liver cells: oxidative DNA damage (ODD), LPO byproduct aldehyde-induced DNA adducts, and AFB1-E-DNA adducts. To test this possibility we used two DNA repair enzymes, formamidopyrimidine DNA glycosylase (Fpg), which specifically recognizes the primary oxidative DNA adduct 8-oxo-dG, and UvrABC, which recognizes bulky DNA damage such as AFB1-E-dG adducts, to determine whether these DNA damages occur in the genomic DNA of AFB1 treated human cells [26]. The results in Figure 1A show that genomic DNA from AFB1 treated hepatocytes are sensitive to UvrABC incision but not to Fpg or endonuclease III (Endo III). Endo III recognizes abasic sites [27]. Therefore, these results indicate that AFB1 induces mainly bulky DNA damage but not purine imidazole-ring open adducts, ODD or abasic sites in liver cells. In contrast, AFB1-E treatment induces UvrABC sensitive sites, as well as Fpg and Endo III sensitive sites in the genomic DNA. It has been found that AFB1-E-dG can spontaneously transform into AFB1-formamidopyrimidine-dG (AFB1-FaPy-dG) adducts which can be depurinated to generate abasic sites [28]. AFB1-FaPy-dG adducts and depurinated sites are sensitive to UvrABC and Fpg and Endo III, respectively [28]. Together, these results also raise a puzzling possibility that AFB1 induced bulky DNA adducts in hepatocytes are not AFB1-E-dG adducts since they are not sensitive to Fpg, indicating that they do not spontaneously transform into AFB1-FaPy-dG. AFB1 treatment does not induce Fpg, Endo III, or UvrABC sensitive sites in the genomic DNA of lung fibroblasts, indicating that these cells are unable to metabolize AFB1 [28].

### AFB1-induced cyclic α-methyl-γ-hydroxy-1,*N^2^*-propano-dG (meth-OH-PdG) adducts in hepatocytes

We then searched for AFB1-induced bulky DNA adducts that are sensitive to UvrABC nuclease and identified AFB1-induced DNA damaging agents other than AFB1-E that resulted from AFB1 treatment in HepG2 cells. We found that AFB1 induces ROS in human HepG2 cells but not in lung CCL-202 fibroblasts (Supplementary Figure 2) [29]. Since AFB1-induced DNA damage is resistant to Fpg and Endo III these results indicate that AFB1 induced ROS are most likely taking place in the cytoplasm and do not react with the nuclear genomic DNA to cause ODD (Figure 1A). It is well established that ROS can trigger LPO, and many LPO byproducts such as aldehydes can induce bulky DNA damage in nuclear DNA [30, 31]. Previously, we found that LPO generated aldehydes such as HNE, Acr, Cro, and Acet can induce bulky cyclic 1,*N^2^*-propano-dG (PdG) adducts that are sensitive to UvrABC incision [20, 32]. Using ^32^P post-labeling two-dimensional thin layer chromatography (2D-TLC) and HPLC analysis, we measured three major LPO byproduct aldehyde-induced bulky cyclic 1,*N^2^*-PdG adducts (γ-OH-PdG, meth-OH-PdG and HNE-PdG) in AFB1-treated hepatocytes [33, 34]. We found that AFB1 treatment induced the 6R,8R isoform of cyclic meth-OH-PdG adducts in a dose-dependent manner in HepG2 cells. However, the AFB1 treatment did not induce significant levels of γ-OH-PdG or HNE-PdG (Figures 1B, 1C, and Supplementary Figure 3). The results in Figure 1C also show that both Acet and Cro can induce 6S, 8S and 6R, 8R isomeric meth-OH-PdG adducts. Together, these results indicate that, in liver cells, AFB1 induces LPO, and that Acet and Cro are the major LPO byproducts that induce meth-OH-PdG adducts.

Using an immunochemical method, we determined the meth-OH-PdG and AFB1-E-dG formation in AFB1 treated hepatocytes [20, 35]. The results in Figure 1D & 1E show that AFB1 induces both adducts in a dose-dependent manner in HepG2 cells. It is worth noting that AFB1 induces >30-fold higher levels of PdG adducts than AFB1-E-dG adducts.

### AFB1 induces bulky DNA damage preferentially at 5’-GG- sequences in p53 including at codon 249

**Figure 2:**
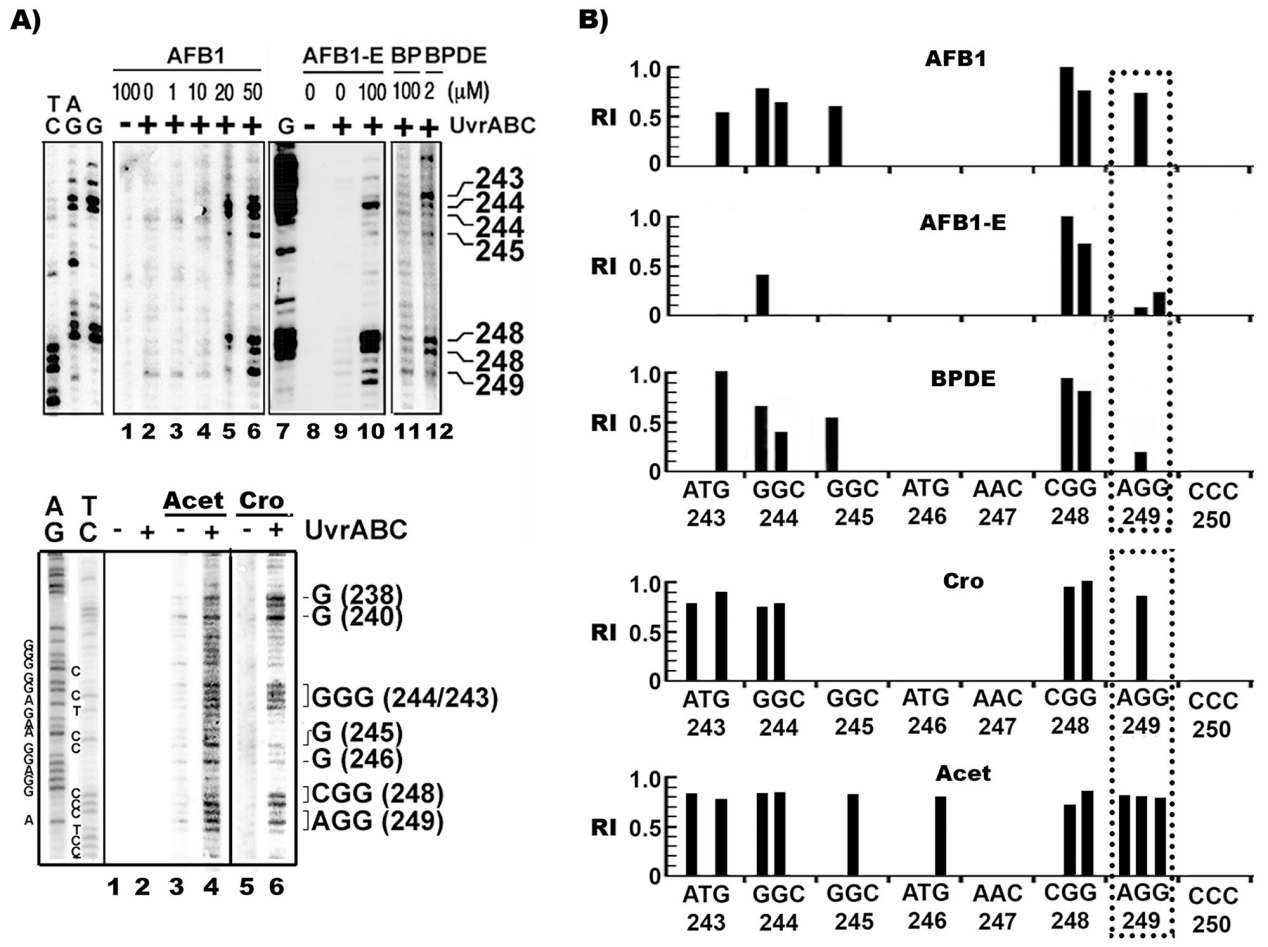
AFB1 as well as Acet and Cro induced bulky DNA damages in human hepatocytes are preferentially formed at 5’–GG- sequences, including the HCC p53 mutational hotspot codon 249 **A**. The bulky DNA damage distributions in the coding strand of exon 7 in HepG2 treated with AFB1, AFB1-E, Acet, and Cro were mapped by UvrABC/LMPCR, as described [36]. The corresponding codon numbers to the UvrABC incision band positions are indicated. Genomic DNA modified with BP diol epoxide (BPDE) *in vitro* (2 μM, 2 h at 37 °C) was included for comparison. AG, TC, and G are Gilbert reaction products [60]. **B**. Relative levels of AFB1, Acet, Cro, AFB1-E, and BPDE induced bulky DNA adducts formed at different sequences of the coding strand of exon 7. The positions of the individual codons are indicated below the sequence. Symbols: +/−, DNA treated with (+) and without (-) UvrABC nuclease. RI represents relative intensity.

The aforementioned results indicate that the major adducts induced by AFB1 treatment in hepatocytes are meth-OH-PdG rather than AFB1-E-dG adducts. These results raise the possibility that the prevalent codon 249 mutations in AFB1 related HCC are due to AFB1 induction of meth-OH-PdG in human liver, which is preferentially formed at this codon. To test this possibility, using the UvrABC incision method in combination with ligation mediated PCR (LMPCR), we mapped the bulky DNA adduct distribution in the p53 gene in hepatocytes treated with AFB1, Cro, Acet, and the AFB1 major metabolite AFB1-E [26, 36]. The results in Figure 2 and Supplementary Figure 4 show that AFB1 induced DNA adducts preferentially formed at 5’-GG- sequences (codons 243, 244, 245, 248, 249, 265, and 267). In contrast, AFB1-E induces DNA damage preferentially at –CpG- sequences including codons 244 and 248 in exon 7 of the *p53* gene (Figure 2A & 2B), which is consistent with our previous results [13]. AFB1 induces bulky DNA damage preferentially at codon 249, whereas AFB1-E induces only modest DNA damage at this codon. Furthermore, we found that Cro and Acet, and AFB induce a similar DNA adduct spectrum in the p53 gene which occurs preferentially at 5’-GG- sites including at codons 226, 243, 244, 245, 248, and 249 (Figure 2A & 2B). Taken together, these results indicate that Acet and Cro are the major DNA damaging agents induced by AFB1 in HepG2 cells, and that the AFB1-induced DNA damage spectrum, including that within the p53 gene, is shaped by these two aldehydes.

### Cytosine methylation at -CG- site enhances Cro-DNA adduction at codon 249 of the *p53* gene

**Figure 3:**
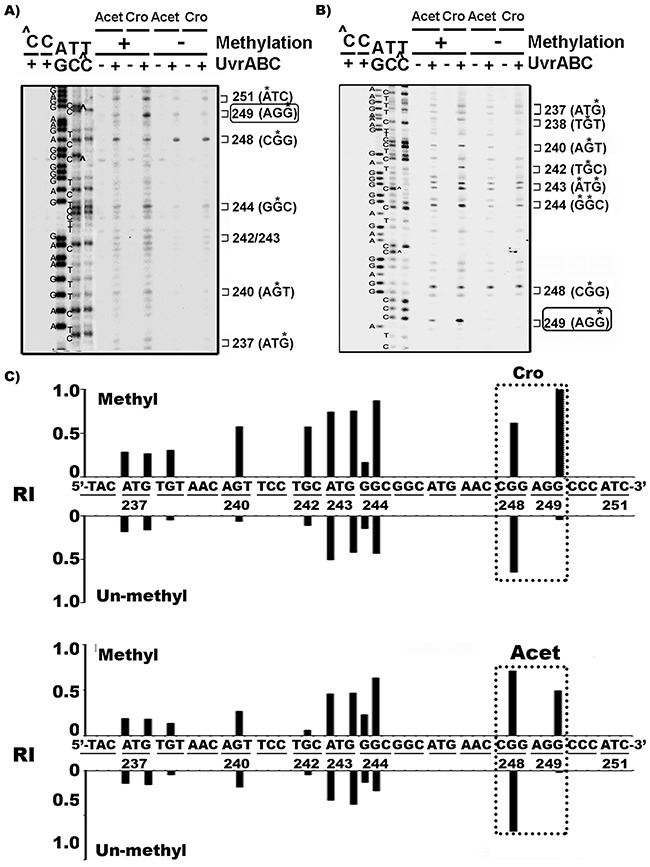
Cytosine methylation at codon 248 (-CGG-) of *p53* gene sensitizes codon 249 (-AGG-) for Acet and Cro modifications Single 5’-end **A**. or 3’-end **B**. ^32^P labeled exon 7 p53 DNA fragments were methylated with CpG methylase, as previously described [13, 61] then modified with Acet (100 μM, 1 h at 25 °C) and Cro (100 μM, 1 h at 37°C). PdG distributions in the fragments were mapped by the UvrABC incision method [26]. ∧C and C represent the methylated and unmethylated DNA fragments, respectively. * indicates the UvrABC incised base. **C**. The effect of methylation on Acet and Cro induced PdG formation. The relative levels of PdG formation at different sequences of 3’-end ^32^P-labled exon 7 of *p53* gene fragments with (Methyl) and without (Un-methyl) CpG methylation as shown in (B) were quantified as described [13, 36]. RI represents relative intensity. Note: CpG methylation greatly enhances Acet- and Cro-induced PdG formation at codon 249.

To determine whether sequence context and/or epigenetic modifications lead to the preferential binding of Acet and Cro at codon 249 in the *p53* gene in AFB1, Acet- and Cro- treated HepG2 cells, we mapped the meth-OH-PdG distribution in Acet- and Cro-modified PCR amplified DNA fragments of exon 7 of the *p53* gene, with and without cytosine methylation at -CG- sites. It has been established that cytosine methylation at CpG sites affects DNA adduct formation at the CpG sites as well as at the neighboring DNA sequences [13, 37, 38]. The results in Figure 3 show that Acet and Cro induced very little, if any, meth-OH-PdG adducts at codon 249 (-AGG-)in unmethylated DNA fragments. In contrast, both Acet and Cro induced high levels of meth-OH-PdG adducts at codon 249 in 5’-CG- methylated DNA fragments. Methylation at 5’-CG- sites did not affect Acet- and Cro-induced PdG formation at the codon 248 (-CGG-); however, methylation at the 5’-CG-sites does enhance meth-OH-PdG formation at other sequences and the observed effect was particularly dramatic at codon 249. These results indicate that cytosine methylation at the 5’-CG- site of codon 248 sensitizes codon 249 to Acet and Cro modifications to form meth-OH-PdG adducts.

### Repair of AFB1-induced meth-OH-PdG and AFB1-E-dG adducts in genomic DNA overall and in exon 7 of the *p53* gene

**Figure 4:**
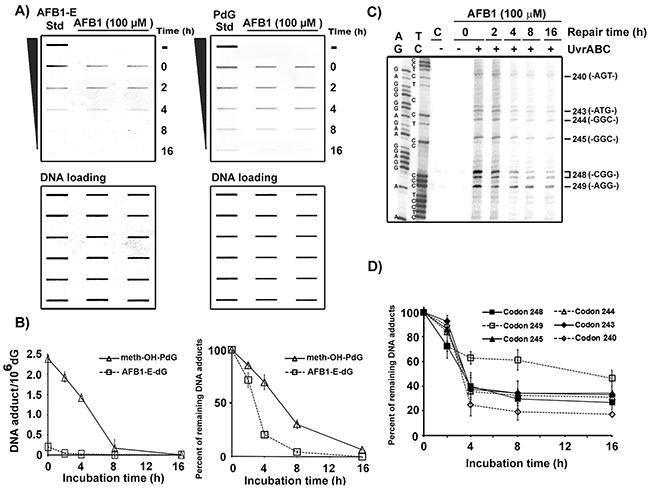
Repair kinetics of AFB1-induced DNA damage in the overall genomic DNA and in the coding strand of p53 gene exon 7 in human hepatocytes HepG2 cells were treated with AFB1 (100 μM, 1 h at 37 °C), then incubated for different time periods (0, 2, 4, 8, and 16 h). **A**. PdG and AFB1-E-dG adducts formed in the genomic DNA were detected by antibody and quantum dot labeling method [20, 33, 34]. AFB1-E or PdG standards (Std) are included for comparison. Amount of input DNA stained by methylene blue. **B**. Repair kinetics of PdG adducts and AFB1-E-dG adducts in the overall genomic DNA. **C**. Mapping DNA damage distribution at sequence level in the coding strand of p53 exon 7 by the UvrABC/LMPCR after different repair times, as described in Figure 2 [36]. **D**. Repair kinetics of DNA damage formed in codons 240, 243, 244, 245, 248, and 249. Note: AFB1-E-dG repair is faster than meth-OH-PdG repair. Repair of meth-OH-PdG in codon 249 is poorer than in other codons.

We then used antibodies that are specific for PdG and AFB1-E-dG to determine the repair kinetics of these two types of DNA adducts induced by AFB1 in the hepatocytes. The results in Figure 4A & 4B show that the repair kinetics of AFB1-E-dG adducts are faster than for meth-OH-PdG adducts in the AFB1-treated HepG2 cells. Using the UvrABC incision method in combination with LMPCR we then determined the repair kinetics of AFB1-induced DNA damage at the coding strand (non-transcribed strand) of the *p53* gene exon 7 in hepatocytes. Figure 4C & 4D show that the repair of DNA damage induced by AFB1 at codon 249 is much slower and less efficient than the repair of adducts formed in the genomic DNA overall and specifically in codons 240, 243, 244, 245, and 248. The repair kinetics of DNA damage formed at codons containing a –CpG- sequence (codons 243, 244, 245, and 248) is slower than at a codon without a -CpG- sequence (codon 240). These results are consistent with our previous finding that the repair of bulky DNA damage formed at –CpG- sites is slower than repair of bulky DNA damage formed at the non-CpG sites [39].

### Mutagenicity of Acet and Cro-induced PdG

**Figure 5:**
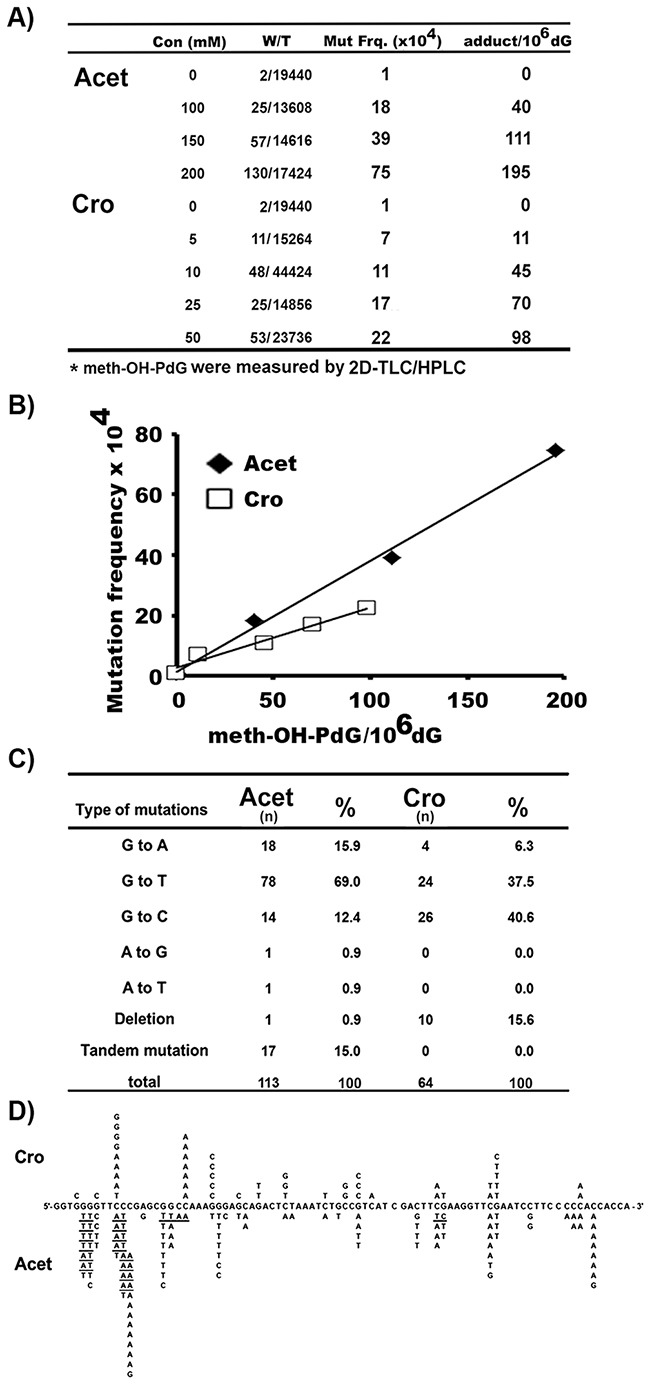
Mutation frequency, spectrum, and signatures induced by Cro- and Acet-DNA modifications Plasmid pSP189 DNAs containing the *supF* gene were modified with different concentrations of Cro and Acet, as described [12, 20, 34]. **A. & B**. PdG DNA adduct levels were determined by the ^32^P post-labeling/2D-TLC/HPLC method, as in Figure 1. Mutation frequencies were detected, as previously described [20, 33, 34]. (A) Mutants and meth-OH-PdG adduct levels induced by different concentrations of Acet and Cro used for the *supF* plasmid modifications. Symbols: Con: concentration; W/T: white colony/total colony; mut frq: mutation frequency. (B) Mutation frequency versus number of Acet- and Cro-induced meth-OH-PdG adducts in plasmid DNA. **C. & D**. Mutational spectrum and signatures induced by Cro- and Acet-modifications. n represents number of mutations. Underlines represent tandem mutations. Note: The percentages of G to T and A mutations induced by Acet are similar to that of induced by AFB1 [40–42].

The Results in Figure 1 show that Cro and Acet induce different ratios of meth-OH-PdG isoforms in hepatocytes, while Cro induces mainly the 6S,8S form of α-meth-γ-OH-PdG, Acet, similar to AFB1, induces mainly the 6R,8R form of α-meth-γ-OH-PdG. We then determined the mutagenicity of the meth-OH-PdG adducts induced by these two aldehydes. Shuttle vectors containing the *supF* gene were modified with different concentrations of Cro and Acet to generate different levels of meth-OH-PdG in the plasmids, which were determined by ^32^P post labeling 2D-TLC/HPLC assay (Figure 1B) [20, 33, 34]. The modified plasmid DNAs were then transfected into HepG2 cells. Mutations induced by meth-OH-PdG adducts were manifested in the *E. coli* indicator cells (M7070) and the types of mutations were determined by DNA sequencing [20, 34]. The results in Figures 5A & 5B show that the meth-OH-PdG adducts induced by Cro and Acet are mutagenic; while the mutation frequency is linearly proportional to the levels of PdG induced by Cro and Acet the Acet-induced meth-OH-PdGs were 2-fold more mutagenic than the Cro-induced meth-OH-PdGs. These results indicate that the (6R,8R)-α-meth-γ-OH-PdG adducts are more mutagenic than (6S,8S)-α-meth-γ-OH-PdG adducts. Furthermore, we found that the mutational spectrum and types of mutations induced by these two aldehydes were distinctly different (Figure 5C & 5D). Most notably Acet induced mainly G to T transversion mutations (69%), which are the same as AFB1-induced G to T mutations in the p53 and *gpt* genes in human cells and yeast systems [40–42]. Cro, on the other hand, induced both G to T (37.5%) and G to C (40.6%) mutations at similar levels [43]. However, the mutational hotspots induced by both Cro and Acet in general are within the same sequence context (at a run of G’s). These results are consistent with the results in Figures 2 & 3, which show that Cro and Acet have the same sequence preference for DNA binding.

### AFB1 treatment inhibits DNA repair

**Figure 6:**
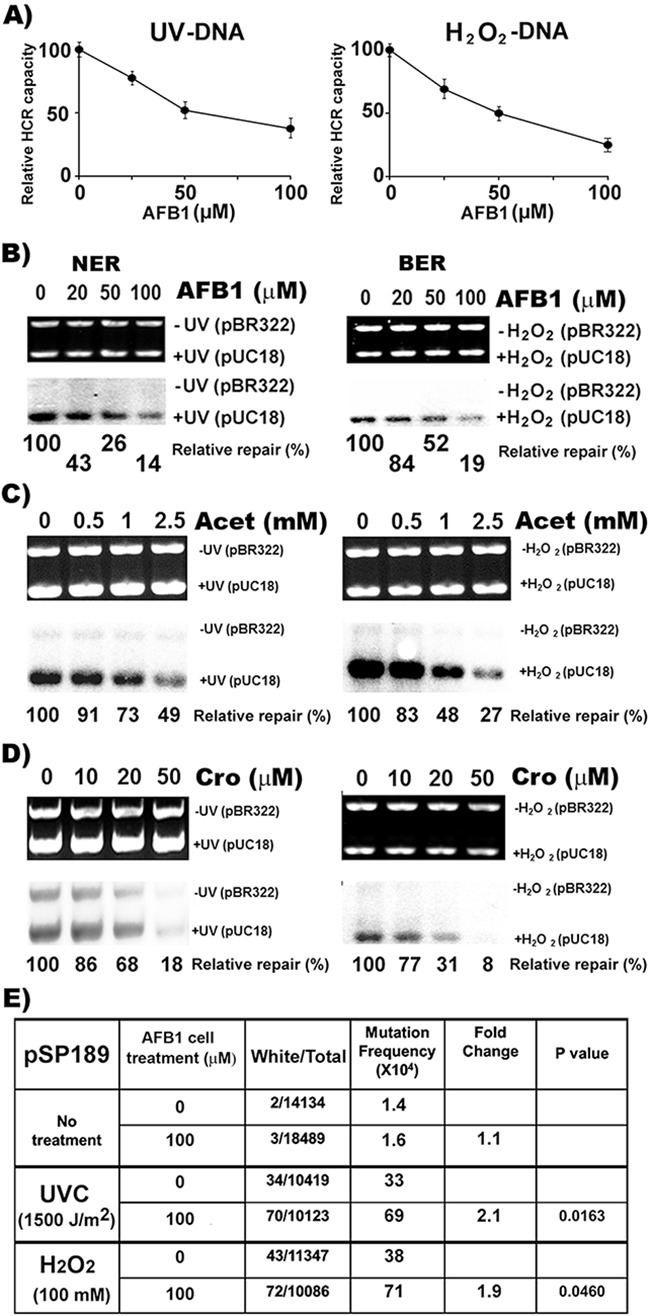
AFB1 treatment inhibits NER and BER capacity and sensitizes cell susceptibility to DNA damage-induced mutagenesis NER and BER capacities were determined by **A**. the host cell reactivation (HCR) assay, and **B, C**. and **D**. the DNA- damage-dependent repair synthesis assay. AFB1, Acet and Cro treatments of HepG2 cells were the same as described in Figures 1, 2, 4 & 5. Methods for the HCR and repair synthesis assays were previously described [12, 20]. (A) The effects of AFB1 treatment on repair capacity for UV- and H_2_O_2_-induced DNA damage are shown. (B, C and D) Representative photograph of ethidium bromide-stained gel (upper panel) and the autoradiograph of the same gel (lower panel). The relative repair capacity calculation is based on the ratio of the amount of repair synthesis over the amount of substrate DNA and is depicted at the bottom of the gels. Note: AFB1, Acet and Cro treatments cause reductions of NER and BER capacity. In **E**. UV- and H_2_O_2_-modified *supF*-containing pSP189 plasmid DNAs were transfected into HepG2 cells with and without pretreatment of AFB1 (100 μM, 6 h at 37 °C). Mutations in the *supF* gene were detected and calculated the same as described in Figure 5 [20, 33, 34]. Note: AFB1 pretreatment enhances mutation susceptibility of HepG2 cells by 2 fold.

We have shown that the major aldehydes generated by LPO, such as malondialdehyde, Acr, and HNE, can not only damage DNA, but can also have an inhibitory effects on nucleotide excision repair (NER), base excision repair (BER), and mismatch repair [12, 20, 21, 44]. We have found that Acr can modify both NER and BER proteins through carbonylation and Michael addition causing DNA repair dysfunction [20]. It is possible that AFB1 may affect NER and BER via Acet and Cro induced effects in AFB1 treated HepG2 cells. Using the host cell reactivation (HCR) assay [12], we tested the effect of AFB1 treatment on NER and BER capacity in HepG2 cells. We found that the expression of UV-irradiated and H_2_O_2_ modified luciferase genes was much lower in AFB1-treated HepG2 cells than in untreated HepG2 cells and that the extent of reduction in reactivating luciferase expression in AFB1-treated hepatocytes was proportional to the concentration of AFB1 (Figure 6A). These results indicate that AFB1 inhibits cellular NER and BER in liver cells. This conclusion was supported by results demonstrating that the cell lysates isolated from AFB1-treated cells have significantly less capacity to carry out UV-irradiation- or H_2_O_2_-modification-induced-DNA repair synthesis than lysates of untreated cells, and again, that these repair synthesis reductions are proportional to the AFB1 concentrations (Figure 6B). The effect of AFB1-induced reduction of cellular repair capacity was not due to the physical interaction of AFB1 with repair proteins, since adding AFB1 directly to cell lysates had no effect on UV-induced DNA repair synthesis. However, adding Acet and Cro to cell lysates instantaneously inhibits NER (Supplementary Figure 5). These results indicate that Acet and Cro can modify repair proteins to cause DNA repair dysfunction, and raise the possibility that the inhibitory effects on NER and BER by AFB1 is due to Acet and Cro induction via LPO. If this is true, we expect that HepG2 cells treated with Acet and Cro should have similar outcomes to those treated with AFB1. Indeed, Acet and Cro treatment similarly inhibits both NER and BER in the HepG2 cells (Figure 6C & 6D).

### AFB1 treatment enhances cell susceptibility to DNA damage-induced mutagenesis

The results presented above demonstrate that AFB1 induces LPO in HepG2 cells and that the LPO byproducts, Acet and Cro, are not only able to induce DNA damage, which preferentially occurs at codon 249 of the *p53* gene, but they also can inhibit both NER and BER. These results raise the possibility that AFB1 can enhance HepG2 cells’ susceptibility to DNA damage-induced mutagenesis. To test this possibility using the *supF* gene containing shuttle vector system, we determined the effect of AFB1 treatment on UV and H_2_O_2_ induced mutagenesis in HepG2 cells [33, 34]. The results in Figure 6E show that UV- and H_2_O_2_-damaged DNA induces 2 fold more mutations in AFB1-treated HepG2 cells than in control cells. These results indicate that AFB1 treatment sensitizes HepG2 cells to be more susceptible to DNA damage induced mutagenesis.

## DISCUSSION

It has long been recognized that AFB1 related HCC has the unique feature of a high level of prevalent mutations at codon 249 of the *p53* gene (>60% of total p53 mutations) [45]. Since the major metabolite in human and rat liver of AFB1 is AFB1-E, which can induce potently mutagenic AFB1-E-dG and AFB1-FaPy-dG adducts, it is generally believed that AFB1-E-induced DNA damage and mutations initiate hepatocarcinogenesis [15, 16]. Indeed, AFB1-E can induce DNA damage and mutations at codon 249, as well as at codon 244, 248, and other codons in the *p53* gene with similar efficiency [45]. However, all of these mutations are also found in HCC that are not AFB1 related, indicating that these mutations can also initiate hepatocarcinogenesis in the same manner as mutations at codon 249 [41]. Why do AFB1-related HCC have such a high incidence of p53 codon 249 mutations? Our results show that AFB1 induced much higher levels of meth-OH-PdG adducts than AFB1-E-dG adducts (>30-fold), and that meth-OH-PdG adducts not only preferentially occur at codon 249 of the *p53* gene but that the repair of the DNA damage formed at this codon is much slower and less complete than repair of DNA damage formed at other codons. These results support the possibility that AFB1 induces LPO and that the LPO byproducts, Acet and Cro, are the major etiological agents causing DNA damage and mutations at codon 249 of p53, the mutation hotspot found in AFB1 related HCC (39,45,46). Although both Acet and Cro can react with DNA to form meth-OH-PdG adducts, Acet exclusively induces the 6R, 8R stereo isoform of meth-OH-PdG adducts, the same meth-OH-PdG adducts that are induced by AFB1 in hepatocytes. Cro, on other hand, induces more 6S, 8S stereo isoform of meth-OH-PdG adducts than 6R, 8R stereo isoform of PdG adducts. Therefore, it is likely that Acet is the major LPO byproduct induced by AFB1 in hepatocytes that causes DNA binding, inhibition of DNA repair, and enhanced mutation susceptibility. In other words, Acet is the mediator that, when induced by AFB1, initiates hepatocarcinogenesis. If this conclusion is correct, it provides a very plausible explanation of why Asians have a high prevalence of HCC: because 40-50% of Asians carry a defective ALDH2 allele that is unable to metabolize Acet to harmless acetate [46, 47]. Therefore, AFB1 induces more Acet and consequently more HCC in the mutant ALDH2 allele-carrying Asians than in ALDH2 wild type carrying Caucasians [48]. It is worth noting that we found that AFB1 causes an inhibitory effect on ALDH2 activity (Supplementary Figure 6).

To account for why AFB1 induces much higher levels of meth-OH-PdG than AFB1-E-dG adducts, we proposed that AFB1 metabolism triggers many cycles of LPO propagation and that LPO generates Acet and Cro byproducts at higher levels than the level of the AFB1-E metabolites. The concentrations of Acet and Cro induced by AFB1 are proportional not only to the AFB1 concentration but, more importantly, are also dependent on the antioxidant levels in hepatocytes, which can stop LPO propagation (Supplementary Figure 7).

The *p53* gene with mutations at codon 249 not only exhibits dominant negative effects on transcriptional transactivation, it also inhibits apoptosis while promoting growth in hepatic cells [49–51]. Interestingly, these effects were not observed for *p53* genes with mutations at codons other than 249 [49–52]. It is therefore likely that the combined effects induced by AFB1, such as the sequence specificity of DNA damage, DNA repair inhibition, the enhancement of mutation susceptibility, and the selective advantage of the mutated codon 249 of the *p53*, all lead to mutations at this gene location in AFB1-related-HCC. Our results show that cytosine methylation at the 5’-CG- site in codon 248 greatly sensitizes the dG in codon 249 to form meth-OH-PdG by Acet and Cro. Although the regulation mechanism for cytosine methylation at 5’-CG- sites in the coding sequence of *p53* including at codon 248 is not known, our results nonetheless raise the possibility that cytosine methylation at codon 248 in hepatocytes may contribute to an individual's susceptibility of AFB1-induced liver cancer.

Taken together, our results show that AFB1 treatment induces oxidative stress and subsequently LPO in hepatic cells. This AFB1-induced effect appears to be chemical specific and limited to pro-carcinogens that are metabolized in these cells. We found that hepatic cells treated with BP do not have this response (Figure 2A and Supplementary Figure 4). BP is metabolized by CYP1A1 that is abundant in lung cells but not in hepatocytes [53].

AFB1 is mainly metabolized by CYP1A2, 2D6, and CYP3A4 [23–25]. These enzymes are abundant in liver cells and lung cells [54, 55]. Metabolism of AFB1 by these enzymes can induce ROS and LPO [17, 18]. The major AFB1 LPO target appears to be arachidonic acid [56], however, it is unclear how this process generates Acet and Cro. Both Acet and Cro are able to modify repair proteins instantaneously causing repair dysfunction (Supplementary Figure 5).

In summary, we found that the liver carcinogen AFB1 induces >30 fold more meth-OH-PdG adducts, the major adducts formed by interactions of Acet and Cro and DNA, than AFB1-E-dG adducts in liver cells, that this type of adduct preferentially occurs at codon 249 of the *p53* gene, the sole mutational hotspot in AFB1 related liver cancer, and that repair of DNA damage at codon 249 is slower and less efficient than at other codons. We found that Acet and Cro-induced meth-OH-PdG adducts are mutagenic, inducing G to T and G to A mutations similar to mutations found in the *p53* in human HCC. AFB1, as well as Acet and Cro, inhibits DNA repair and enhances cell mutation susceptibility. These results lead us to hypothesize a novel mechanistic process by which AFB1 induces hepatocarcinogenesis (Supplementary Figure 7). Metabolism of AFB1 in liver cells not only induces AFB1-E but it also triggers LPO cycles, consequently generating Acet and Cro which can induce meth-OH-PdG adducts, inhibit DNA repair and enhance cell susceptibility to mutagenesis; and meth-OH-PdG adducts preferentially occur at codon 249 of the *p53* gene of genomic DNA with 5’-CG- methylated at codon 248. Based on this knowledge, we propose that AFB1-related HCC can be prevented by antioxidants and sulfhydryl compounds, which can stop the LPO cycle and/or neutralize aldehydes.

## MATERIALS AND METHODS

### Cell growth, carcinogen treatment and genomic DNA isolation

Human hepatocytes HepG2 and lung fibroblasts CCL-202 (American Type Culture Collection, Manassas, VA) were grown in Eagle's minimum essential medium (Sigma-Aldrich, St. Louis, MO 63178 USA) supplemented with 10% fetal bovine serum. At 70% confluency, cells were treated for varying durations with different concentrations of AFB1, BP, Cro, Acet (Sigma-Aldrich, St. Louis, MO 63178 USA), or AFB1-E which was prepared as described. Genomic DNA was isolated as previously described [12].

### DNA adducts analysis

The formation of AFB1-induced cyclic 1, *N^2^*-propano-DNA adducts in HepG2 cells was detected first by ^32^P post-labeling in combination with 2D-TLC and the DNA adducts were further separated by HPLC using a 10-50% gradient in methanol/water (50:50) buffer [20, 33, 34]. The AFB1-E-dG adduct was detected by competitive ELISA similar to the method previously described [57]. The monoclonal AFB1-E-dG antibody and standard were gifts from Dr. Regina Santella at Columbia University.

### UvrABC, Fpg, and Endo III incision assay and ligation mediated PCR (LMPCR)

UvrABC enzymes were prepared as previously described [58]. Fpg and Endo III were gifts from Dr. Yoke Kow at Emory University. It is well established that the UvrABC nuclease incises bulky DNA damage including PdG, BPDE-dG, and cyclobutane pyrimidine dimers, Fpg incises 8-oxo-deoxyguanines and formamidopyrimidines, and endonuclease III incises thymine glycols and abasic sites [13, 26, 59]. The LMPCR method used for mapping the distribution of bulky DNA adduct formed in the p53 gene is the same as previously described [12, 36].

### Acet and Cro modifications of cytosine methylated and unmethylated DNA fragments

Methods for obtaining 5’ and 3’ single-end ^32^P labeled DNA fragments containing the human *p53* exon 7 sequences were as previously described [13, 36]. DNA fragments were treated with CG methylase to methylate cytosines at 5’-CpG- sites, then modified with Acet (100 mM, at 25 °C, 1 h) and Cro (100 mM, at 37 °C, 1 h) [37]. The PdG distribution is mapped by UvrABC incision method, as previously described [26].

### Host cell reactivation (HCR) and *in vitro* DNA-damage-dependent repair synthesis assay

DNA repair capacity was detected by the HCR assay and by the *in vitro* DNA damage dependent repair synthesis assay as previously described [20].

### Mutagenicity of Acet and Cro-induced PdG adducts

Shuttle vector pSP189 plasmid DNAs containing the *supF* gene were modified with different concentrations of Acet (0, 100, 150, and 200 μM, 1 h at 25 °C) and Cro (0, 5, 10, 25, and 50 μM, 1 h at 37 °C); the PdG formed in the plasmid DNA were detected by slot blot using PdG specific monoclonal antibodies and by ^32^P post-labeling and 2D-TLC/HPLC, as described [20, 33, 34]. Modified plasmid DNAs were transfected into HepG2 cells. The replicated plasmid DNAs were recovered 72 h after transfection by Hirt's method and the mutations in the *supF* gene were detected using the previously described method [20, 33, 34].

### Effect of AFB1 treatment on mutation susceptibility

HepG2 hepatocytes were pretreated with AFB1 (0.1 mM, 6 h) then transfected with of UV (1500 J/m^2^) irradiated or H_2_O_2_(100 mM, 30 min) modified *supF* containing pSP189 plasmid DNA. The mutations in the *supF* gene were detected using the previously described method [20, 33, 34].

## SUPPLEMENTARY MATERIALS FIGURES


