# Supplementary material for: AFB1 hepatocarcinogenesis is via lipid peroxidation that inhibits DNA repair, sensitizes mutation susceptibility and induces aldehyde-DNA adducts at p53 mutational hotspot codon 249
Source: Oncotarget. 2017 Feb 14;8(11):18213–26. doi: 10.18632/oncotarget.15313 (PMC5392321; doi:10.18632/oncotarget.15313)
Supplement: Supplementary file 1 [file oncotarget-08-18213-s001.pdf]

AFB1 hepatocarcinogenesis is via lipid peroxidation that inhibits DNA repair, sensitizes mutation susceptibility and induces aldehyde-DNA adducts at p53 mutational hotspot codon 249

SUPPORTING INFORMATION

REFERENCES

1. Petitjean A, Mathe E, Kato S, Ishioka C, Tavtigian SV, Hainaut P and Olivier M. Impact of mutant p53 functional properties on TP53 mutation patterns and tumor phenotype: lessons from recent developments in the IARC TP53 database. Human mutation. 2007; 28:622-629.
2. Arakawa H, Weng MW, Chen WC and Tang MS. Chromium (VI) induces both bulky DNA adducts and oxidative DNA damage at adenines and guanines in the p53 gene of human lung cells. Carcinogenesis. 2012; 33:1993-2000.
3. Feng Z, Hu W, Hu Y and Tang MS. Acrolein is a major cigarette-related lung cancer agent: Preferential binding at p53 mutational hotspots and inhibition of DNA repair. Proceedings of the National Academy of Sciences of the United States of America. 2006; 103:15404-15409.
4. Shah A, Xia L, Goldberg H, Lee KW, Quaggin SE and Fantus IG. Thioredoxin-interacting protein mediates high glucose-induced reactive oxygen species generation by mitochondria and the NADPH oxidase, Nox4, in mesangial cells. The Journal of biological chemistry. 2013; 288:6835-6848.

SUPPLEMENTARY FIGURES

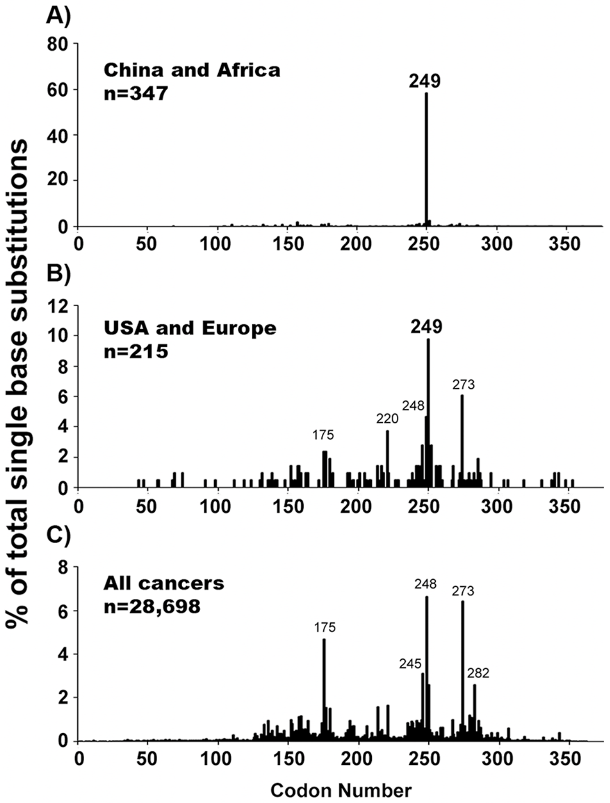

Supplementary Figure 1: The p53 mutational spectrum in **A.** HCC from AFB1 contaminated countries (China and Africa), **B.** HCC from countries without AFB1 food contamination (USA and Europe), and C. all human cancer. The data were obtained from the *p53* mutation data base [1].

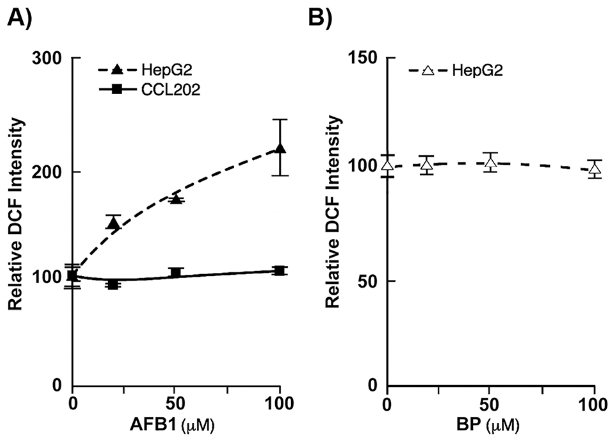

Supplementary Figure 2: The effects of **A.** AFB1 and **B.** BP on reactive oxygen species (ROS) production in human hepatocytes HepG2 (A & B) and human lung fibroblasts CCL-202 A. Cells were incubated with various concentrations of AFB1 or BP for 6 h at 37 °C, and the ROS response was measured using 2',7'-dichlorofluorescin diacetate (DCFH-DA). Relative ROS production (percent control) is expressed as the ratio of fluorescence (dichlorofluorescin [DCF] intensity) of the treated samples versus the response in the appropriate controls. The values represent the average of three independent experimental results and the bars represent standard deviations.

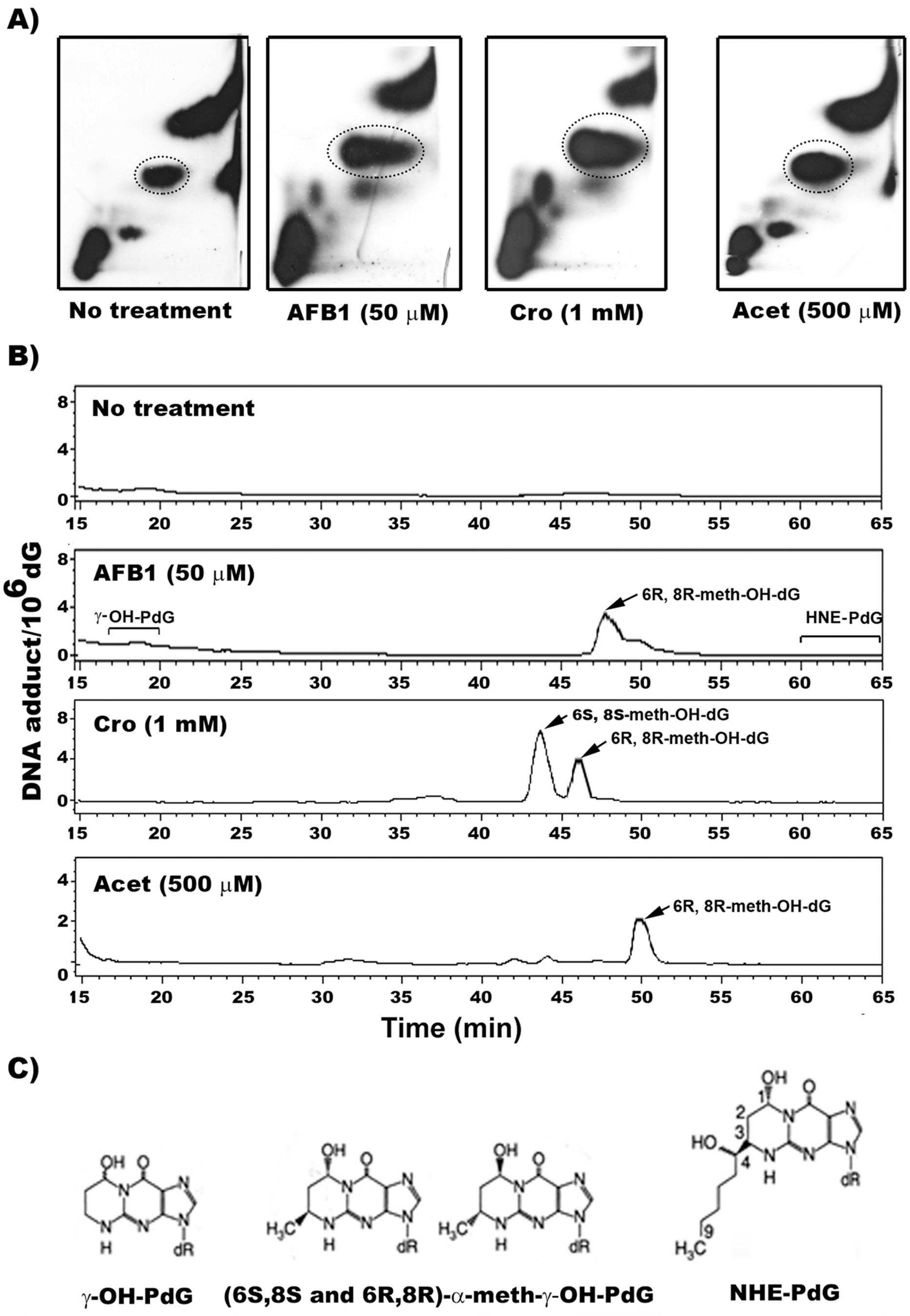

Supplementary Figure 3: DNA adduct analysis of genomic DNA of hepatocytes HepG2 treated with AFB1, crotonaldehyde (Cro) and acetaldehyde (Acet). A. Autoradiographs of 2D-TLC and **B.** chromatograms of HPLC analysis. Genomic DNAs isolated from HepG2 cells treated with AFB1, Cro and Acet were subjected to ^{32}P post-labeling and 2D-TLC followed by HPLC analysis the same as shown in Figure 1. (A) Typical 2D-TLC autoradiographs. The circled spots were eluted and subjected to HPLC analysis. (B) Typical HPLC elution profiles. The elution peak positions of cyclic propano-dG, γ-OH-PdG, meth-OH-PdG and HNE-PdG induced by Acr, Cro, Act and HNE were indicated. Note: AFB1 induces meth-OH-PdG adducts which co-elute with Cro- and Acet-induced 6R, 8R-meth-OH-PdG adducts. **C.** Chemical structures of γ-OH-PdG, meth-OH-PdG and HNE-PdG.

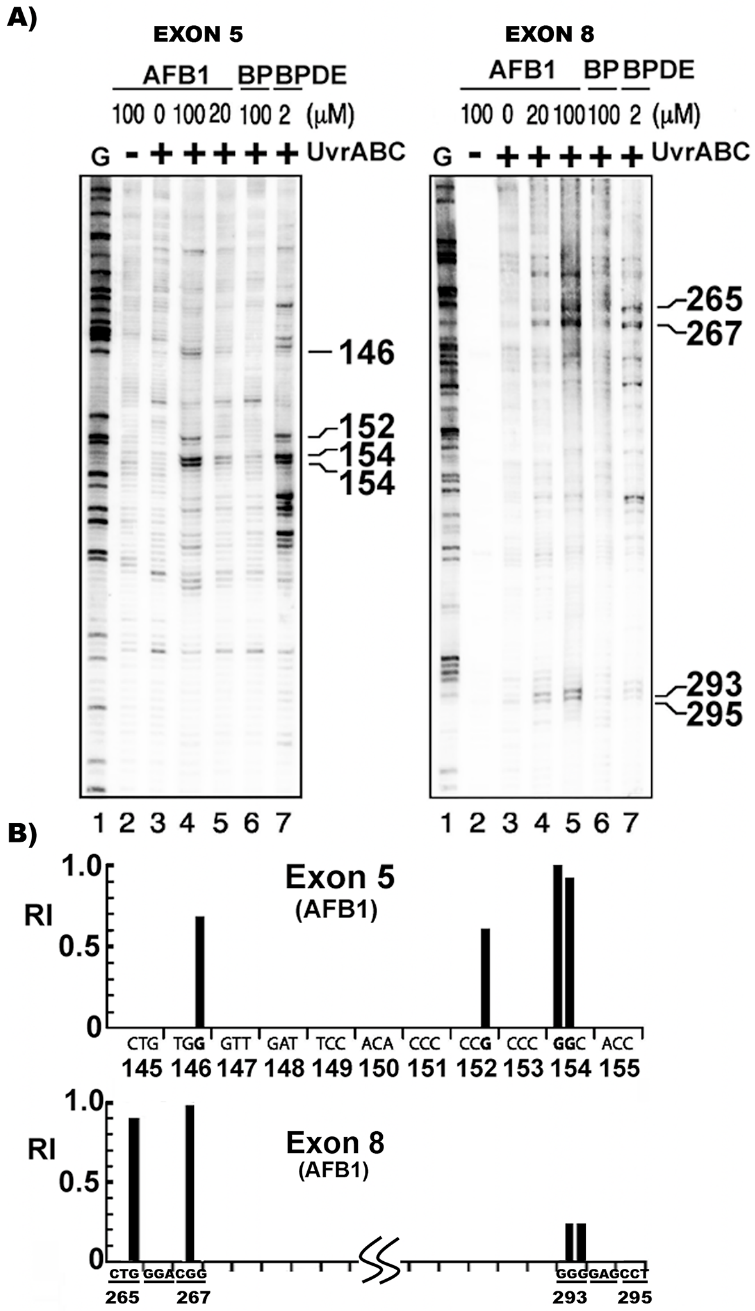

Supplementary Figure 4: The bulky DNA damage distributions in the coding strand of exon 5 and 8 of the p53 gene in HepG2 cells treated with AFB1, BP, and BPDE. The bulky DNA adducts were mapped by the UvrABC incision and ligation mediated PCR (LMPCR) method as described in Figure 1 and [2, 3]. **A.** Typical autoradiographs of the UvrABC-LMPCR products separated by sequencing gels. Bands represent the bulky DNA adducts incised by UvrABC. The codon positions are indicated. **B.** Relative levels of AFB1 induced bulky DNA adducts formed in the coding strand of exon 5 and 8. The positions of the individual codons are indicated below the sequence. Symbols: +/-, DNA treated with (+) and without (-) UvrABC enzymes. RI: relative intensity of UvrABC incision bands.

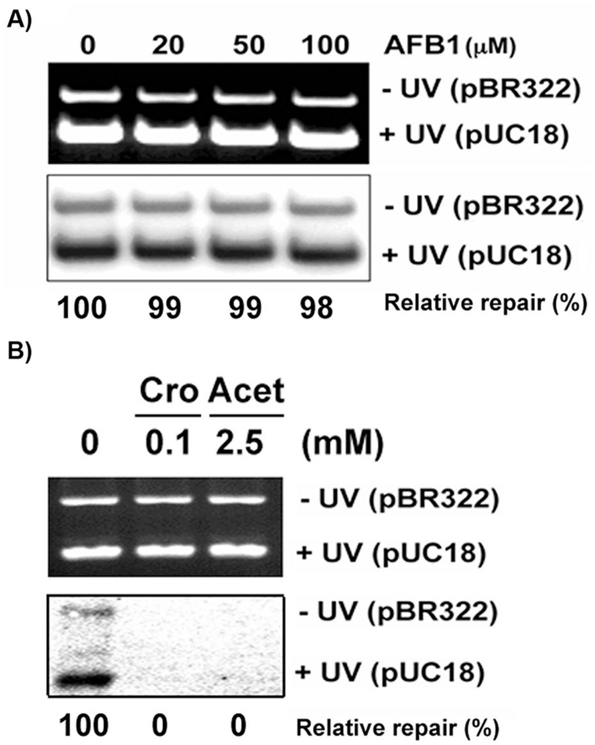

Supplementary Figure 5: Effects of AFB1, Acet and Cro on DNA repair activity in cell- free cell lysates. Different concentrations of **A.** AFB1, and **B.** Acet and Cro were directly added to cell-free cell lysates prepared from control HepG2 cells and the DNA-damage-dependent-repair synthesis was performed as in Figure 6. The relative repair capacity was calculated based on the ratio of the amount of DNA repair synthesis over the amount of substrate DNA and the value in the control sample as 100%. Note: Acet and Cro inhibit DNA-damage-dependent repair synthesis in cell-free cell lysates, whereas AFB1 does not.

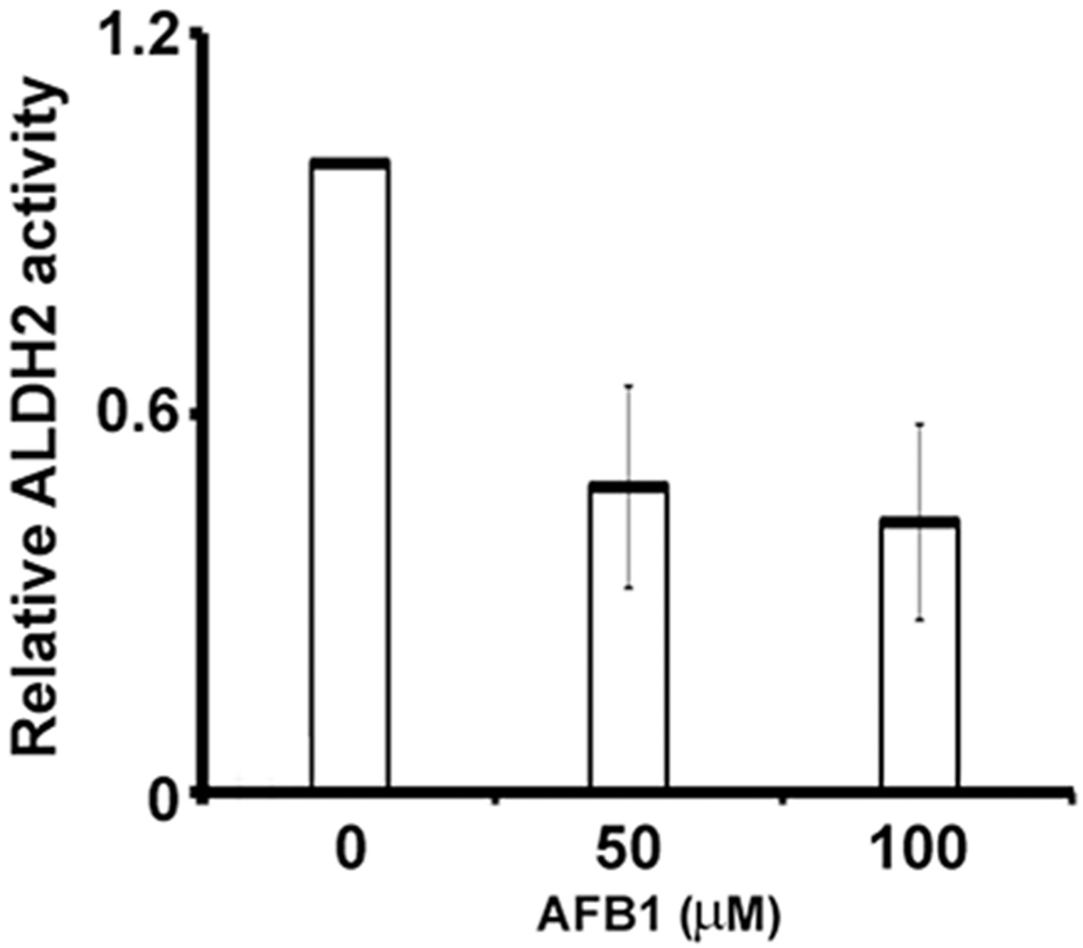

Supplementary Figure 6: AFB1 treatment inhibits acetaldehyde dehydrogenase (ALDH2) activity. Exponentially growing HepG2 cells were treated with different concentrations of AFB1 (0, 50 and 100 μM, 6 h). The activity of ALDH2 was determined as described [4]. (0 vs. 50 μM, p<0.05; 0 vs. 100 μM, p<0.005)

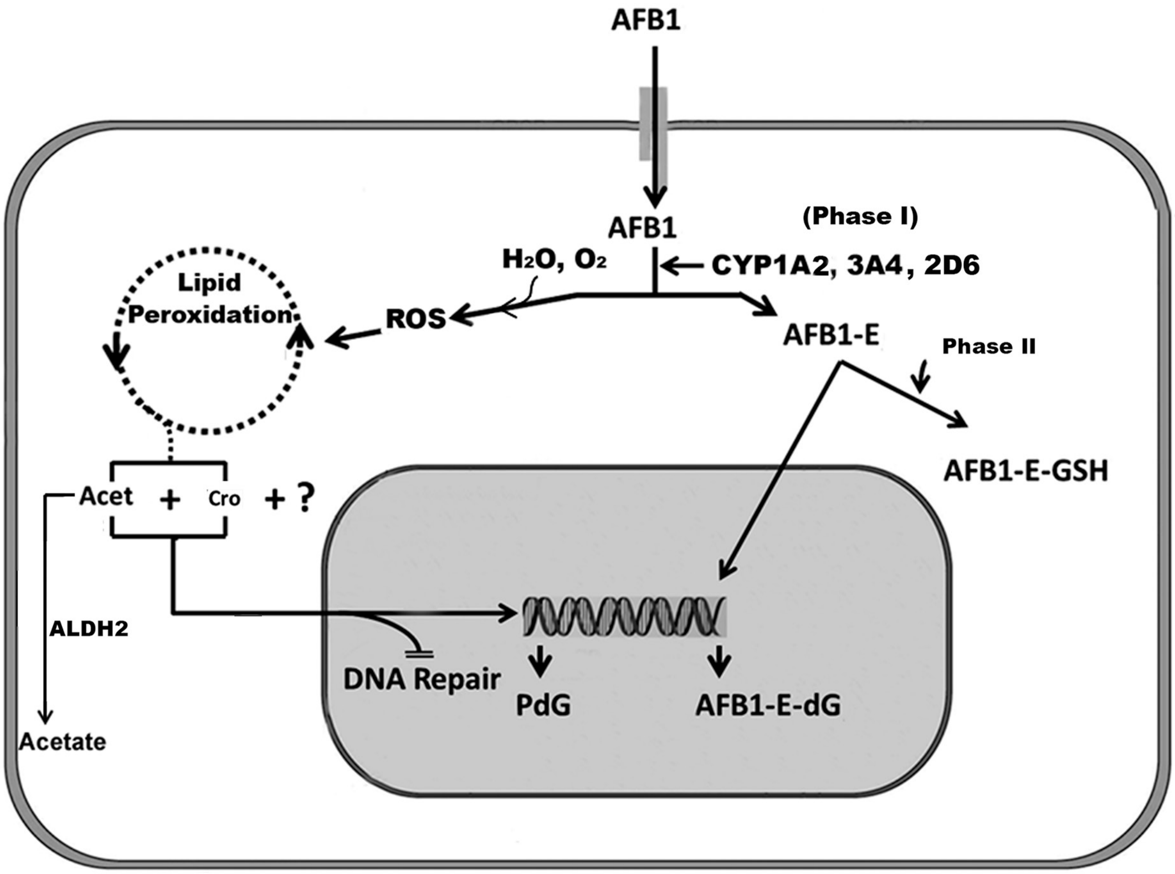

Supplementary Figure 7: A proposed model on AFB1 processing, DNA adduct induction and DNA repair inhibition in human hepatocytes. AFB1 metabolism induces AFB1-E and initiates LPO cycles. AFB1-E may conjugate with glutathione (GSH) or react with genomic DNA to form AFB1-E-dG adducts. LPO produces by-products Acet and Cro which can react with genomic DNA to form meth-OH-PdG adducts and react with repair proteins to inhibit DNA repair functions.
